# The Evolutionary Radiation of Hominids: a Phylogenetic Comparative Study

**DOI:** 10.1038/s41598-019-51685-w

**Published:** 2019-10-24

**Authors:** Guido Rocatti, S. Ivan Perez

**Affiliations:** División Antropología, Facultad de Ciencias Naturales y Museo, Universidad Nacional de La Plata, CONICET, La Plata, Argentina

**Keywords:** Biological anthropology, Palaeontology

## Abstract

Over the last 150 years the diversity and phylogenetic relationships of the hominoids have been one of the main focuses in biological and anthropological research. Despite this, the study of factors involved in their evolutionary radiation and the origin of the hominin clade, a key subject for the further understanding of human evolution, remained mostly unexplored. Here we quantitatively approach these events using phylogenetic comparative methods and craniofacial morphometric data from extant and fossil hominoid species. Specifically, we explore alternative evolutionary models that allow us to gain new insights into this clade diversification process. Our results show a complex and variable scenario involving different evolutionary regimes through the hominid evolutionary radiation –modeled by Ornstein-Uhlenbeck multi-selective regime and Brownian motion multi-rate scenarios–. These different evolutionary regimes might relate to distinct ecological and cultural factors previously suggested to explain hominid evolution at different evolutionary scales along the last 10 million years.

## Introduction

The origin and evolution of humans have been the main focus of biological and anthropological research during the last 150 years^1–3^. Most of the previous studies have focused on species diversity and the phylogenetic history of humans and its relatives (i.e., the hominids^4–6^). Particularly, during the last years the studies on these topics have thrown a relatively large species diversity within Hominoidea^7^ (but see ref.^8^) and a well-supported pattern of phylogenetic relationships for both extant and fossil species^9–11^. However, less attention has been placed to formally explore the factors involved in the hominid evolutionary radiation^12^.

Previous studies have shown that along its evolutionary radiation, hominids have mainly diversified in body size, locomotor apparatus and cranial size and shape^3,13–15^, together with ecological and behavioural characteristics^12,16^. Within this radiation, humans differentiated considerably from the rest of the species mainly in cranial size and shape (e.g. refs^13,15^). In this context, it has been suggested that the hominids, and particularly the hominins, experimented one or several adaptive radiations —i.e., the rapid diversification of an ancestral species in morphologically and ecologically diverse ones^16–18^—, in contrast with the more traditional view of a continuous and gradual process where *Homo sapiens* was the last stage^12–14,19,20^.

Changes in cranial size and shape were important for the evolutionary radiation of a clade as Hominoidea because they are believed to be related to numerous aspects of primate biology. Particularly, variation in ecological and behavioural characteristics —such as locomotion, diet or social organization— is related to the variation in specific cranial traits in the clade^21^. Specifically, in Primates there seems to be a close relationship between the locomotion and the relative position of the foramen magnum, the diet and the size and shape of the arcade, the social group size and the size and shape of the brain endocast, among others^22–26^. In this context, studies interested in the evolutionary radiation of hominids have hypothesized some of these behavioural or ecological factors as the responsible for, or related with, the morphological changes observed in their crania^12,16^.

Here we approach the evolutionary radiation of hominids by analyzing extant and fossil species of this family. We explore alternative models on hominid diversification in cranial size and shape, employing 3D images, geometric morphometric and phylogenetic comparative methods^27–30^. Specifically, we fit random and non-random evolutionary models to the morphometric data, testing for a continuous-gradual (Brownian motion; BM) versus a non-gradual radiation process in the clade (Ornstein Uhlenbeck; OU)^31^. Our approach combines a detailed description of morphometric changes with a rigorous evaluation of alternative evolutionary models including extant and fossils species and using a maximum-likelihood model selection framework. The main goal of this work is to gain new insights into the pattern of cranial diversification in hominid’s —with special emphasis on hominins— evolutionary radiations by using a full quantitative approach, and to compare the magnitude and pattern of variation with the closest relative clade, Hylobatidae family.

## Results

### Morphometric variation among species

**Figure 1:**
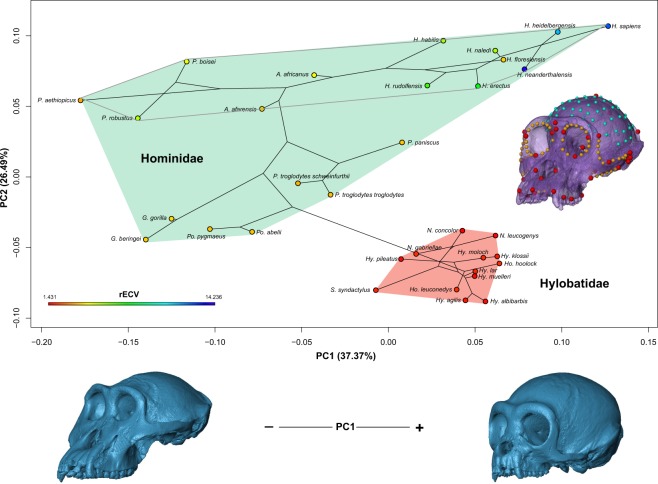
Morphometric analyses of hominoid primate’s craniofacial shape. (Top) Ordination of 21 extant and 12 fossil hominoid species in the phylomorphospace determined by the first two principal components (PC1 and PC2) of craniofacial variation. Together, they account for ~63% of total variance. Subtribe *Hominina* clade is defined by the filled gray line. Cranium at mid-right shows the located landmarks (red), curves (yellow) and surface (cyan) semilandmarks on each individual. Each species endocranial volume relative to its cranial base centroid size (rECV) is defined by the color on each data point. The two hominoid families are indicated. (Bottom) Craniofacial shape changes correspondent to PC1. These were obtained by warping the minimum and maximum PC1 scores in R software (for a visualization of shape changes in PC2 see Fig. S1).

Changes in craniofacial shape in extant and fossil Hominoidea species were studied by means of geometric morphometric techniques^28,30^, using landmarks and semilandmarks on cranial surfaces obtained from computed tomographies (Fig. 1). Landmarks and semilandmarks were aligned with Procrustes Generalized Analysis, in order to obtain shape variables, and the shape differences were explored by means of Principal Component Analyses (PCA), together with the projection of the phylogeny onto the morphospace (henceforth called *phylomorphospace*)^32^. Figure 1 shows the ordination of Hominoidea along the phylomorphospace corresponding to the first two principal components (PC 1 and 2), representing more than 63% of total variation in cranial shape.

A large morphological variation was depicted on such ordination, especially regarding the cranial vault shape, the maxilla length and the proportional size of the orbits and the zygomatic arches. Towards the positive values of PC1 we find specimens with a more globular shape of the cranial vault, together with an increase in the proportional size of the orbits, and a correlated anteroposterior shortening of the facial region —specifically seen as a retrusion of the maxilla— and a reduction of the zygomatic arches relative size (Fig. 1). Hominidae family displays most of its shape variation along PC1. *Homo sapiens* and the rest of the genus *Homo* are located on the positive extreme of this PC; while on the opposite extreme we find *Gorilla* and *Paranthropus* genus, with a more robust craniofacial shape. A clear distinction between Hominidae and Hylobatidae families can be seen along PC2, associated to shape changes in the cranial base and differences in the maxilla orientation (Fig. S1). Hylobatidae family is located towards negative values of PC2, with less flexed crania and less intra-clade variation relative to hominins.

### Phylogenetic pattern of diversification

The colours of the tips in Fig. 1 show the variation in the relative endocranial volume, obtained by mapping species’ endocranial volume values relative to its cranial base centroid size (rECV), depicting that positive values of PC1 and PC2 coincide with higher values of rECV. This correlation was tested by a Phylogenetic Generalized Least Squares analyses (PGLS), showing a low association (*Adjusted R*^2^ = 0.019; *p*-value = 0.210). Moreover, although the phylomorphospace seems to be indicating a lack of influence of size, we performed PGLS analyses in order to test for correlation between these variables^33^. The shape variation of hominoid crania (PC1-9 [~90% of total variation]), as seen in the PGLS results, is not associated to the log centroid size (log CS; *Adjusted R*^2^ = 0.164; *p*-value = 0.0112). This shows that shape variation explained by size is low, ca. 16%, and, thus, points out that the allometric component of shape variation is very small. Particularly, the tendency to a relative increase in the facial skeleton in respect of a larger cranial size, generally observed in mammals, is not observed in the hominoid clade. For example, *Gorilla gorilla*, *Pan troglodytes* and *Homo sapiens* are large species but show different relative sizes of the facial skeleton in regard to the neurocranium, as well as variation in the relative position of both structures.

With the objective of assessing the phylogenetic structure present in the shape and rECV data, we performed phylogenetic signal analyses^34,35^. The results showed high *K* values for PC2 (*K*_*PC2*_ = 1.89; *p* = 0.001), indicating a strong phylogenetic signal, while *K* values for PC1 and rECV were lower (*K*_*PC1*_ = 0.64; *p* = 0.001; K_rECV_ = 0.41 *p* = 0.001). Therefore, shape variation explained by PC2 surpassed what was expected by Brownian motion. This corresponds with the ordination shown in Fig. 1, where PC2 separates Hylobatidae and Hominidae families. The phylogenetic signal analysis for Procrustes coordinates of the aligned *landmarks* and *semilandmarks* showed a low phylogenetic signal (*K*_*mult*_ = 0.29; *p* = 0.001).

**Figure 2:**
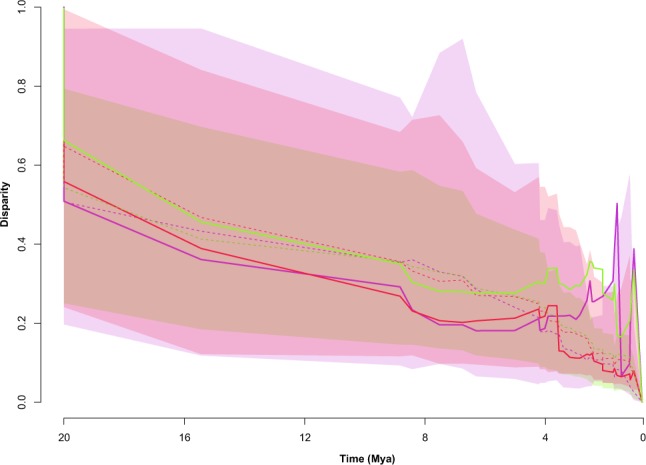
Disparity-through-time (DTT) plot using PC1-2 (red) and PC1-9 (green) scores, estimated from the hominoid species morphometric data, and rECV (purple). Average value at a given point in time is the average disparity of subclades whose ancestral lineages were present at that time relative to the disparity of the entire clade. Higher values of relative disparity correspond to greater variance values within subclades relative to the morphological disparity of the whole subclade. Dashed lines depict the mean of simulated disparity under a Brownian motion model. Shaded areas represent 95% confidence intervals of the simulated data.

To visualize the phenotypic variation pattern of cranial shape and rECV within and among Hominoidea clades through their evolutionary history we used disparity-through-time (DTT)^36^ plots (Fig. 2), employing a chronophylogenetic tree, PC1-9 scores, PC1-2 scores and rECV values. PC1-9 DTT-plot depicts a pattern of progressive increase of among-clade disparity slightly above Brownian motion model expectations, with three main peaks of within-clade disparity growth. The two first of these took place ca. 4-3 million years (myr; also seen in PC1 phenotypic variation pattern; see Fig. S2) and they may coincide with the appearance of australopithecines and the *Paranthropus* species, while the second peak (ca. 1 myr) seems to concur with the shape changes related to the most recent *Homo* species. PC1-2 DTT-plot displays a similar pattern, within values of disparity expected for a BM model, but presenting only a major increase of the among-clade disparity, ca. 4 myr. Moreover, the DTT-plot corresponding to rECV, also follows the trend marked by a BM model expectations, but presenting two main peaks of among-clade disparity between ca. 2–0.5 myr, near the appearance and diversification of *Homo* genus. Figure 2 shows that there might have been a slight decoupling between the disparity patterns for shape and rECV, suggesting that some morphological changes, such as those observed in the facial region, may have occurred earlier (ca. 4–3 myr) than those in the cranial vault related to the increase of rECV.

### An evolutionary scenario for the hominid radiation

**Figure 3:**
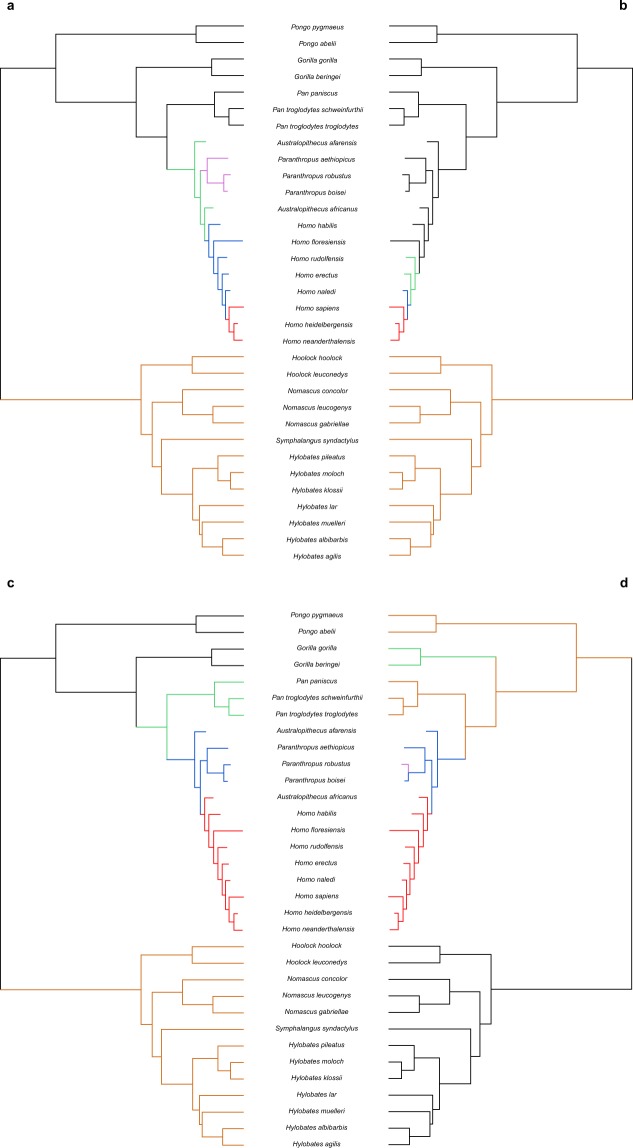
Time-calibrated phylogenetic trees of the Hominoid clade with colored branches, according to the evolutionary hypotheses used for model-fitting tests. (**a**) Foley’s (2002) hypothesis, where each color determines an adaptive radiation. (**b**) Data-driven hypothesis estimated via SURFACE analysis using rECV values. (**c,d**) Data-driven hypotheses estimated via SURFACE analyses using PC1-2 and PC1-9 scores, respectively. Colors in (**b–d**) refer to different adaptive regimes.

These previous results suggest that the studied traits might have evolved mainly following a Brownian motion scenario, despite there seem to have been moments of deviation from the expectations under this model. In order to further elucidate the evolutionary processes driving the morphological diversification in the clade, we compared the fit of several evolutionary models to the PC and rECV data: single-rate Brownian motion (BM), Early Burst (EB) and single-selective regime Ornstein-Uhlenbeck (OU) processes, together with OU and BM multi-selective regime and multi-rate models based on Foley’s^12^ hypothesis about hominid diversification (Foley’s OUM and BMM, respectively)^31,37^. These models allow to explicitly test for a random continuous-gradual (BM) versus a discontinuous radiation (OU) process in the clade based on previous hypotheses. Additionally, we generated data-driven models to better explore the characteristics of the hypothesized discontinuous OU process, employing the SURFACE approach^38^ (Fig. 3). All the models were fitted to the datasets employing the multivariate functions of *mvMORPH* package^37^. By means of Likelihood and Akaike Information Criteria (AIC and AICc), used to select the best evolutionary model, we concluded that Foley’s successive radiations hypothesis following BMM and OUM models showed a better fit than the simplest model without shifts (Table 1). However, SURFACE-based scenarios (Surface’s OUM and BMM) depicted slightly better fits than Foley’s model. Specifically, PC1-2 and rECV SURFACE hypotheses showed their best fit for a multi-selective regime OU process (AICc_PC1-2_ = −300.418; AICc_rECV_ = 71.177). This suggests that there is some relevant aspect in our data that it is not entirely explained by Foley’s hypothesis or the scenario without shifts. Additionally, the PC1-9 dataset displays more variable results, with Likelihood supporting a multi-rate BM model and AIC supporting a multi-rate OU model, whereas AICc values suggest a simple BM model for shape evolution (Table 1). These inconsistencies in the PC1-9 dataset model fit values are probably related to the large number of variables in relation to the number of species studied.

**Table 1:** Results of the multivariate model-fitting analyses for craniofacial shape and rECV values.

			Model	log Likelihood	AIC	AICc
PC1 + PC2	Hypothesis based	Simple hypotheses	BM1	125.0221	−240.0442	−239.0442
			EB	125.0221	−238.0442	−236.6204
			OU1	128.9203	−237.8407	−233.8407
		Foley (2002)	BMM	146.3449	−252.6897	−234.023
			OUM	**171.5918**	**−303.1835**	**−284.5168**
	Surface		BMM	116.8672	−199.7344	−186.9844
			OUM	**175.4859**	**−314.9718**	**−300.4187**
rECV	Hypothesis based	Simple hypotheses	BM1	−80.9682	165.9364	166.3364
			EB	−80.9682	167.9364	168.7640
			OU1	−74.5763	157.1526	158.5811
		Foley (2002)	BMM	**−30.4036**	**74.8072**	**79.2872**
			OUM	−34.5514	87.1028	94.9289
	Surface		BMM	−36.3884	84.7767	88.0075
			OUM	**−24.5886**	**65.1772**	**71.1772**
PC90%	Hypothesis based	Simple hypotheses	BM1	589.7973	−1071.595	**−1047.049**
			EB	586.9523	−1063.905	−1038.344
			OU1	684.0796	−1152.159	−1026.925
		Foley (2002)	BMM	**790.5879**	−1023.176	8167.412
			OUM	735.1081	**−1164.216**	−834.6778
	Surface		BMM	**790.8873**	−1023.775	8166.814
			OUM	747.605	**−1189.21**	**−859.6715**

Values in bold correspond to the best fitted models for previous hypotheses and for data-driven hypotheses.

## Discussion

In the present work we approached the evolutionary radiation of hominids, exploring alternative models for this clade’s cranial size and shape diversification and testing for a continuous-gradual versus non-random models of diversification in the family. We also compared the disparity of this family relative to their sister family: Hylobatidae. Our morphometric analyses showed a large morphological variation in hominoids regarding the cranial vault shape, the maxilla length and the proportional size of the orbits and zygomatic arches. It is remarkable that hominids display larger dispersion in the shape space than the hylobatids, suggesting the occurrence of more complex processes ‒such as going through more adaptive peaks‒ in the former clade. Analyses of the patterns of diversification on these characteristics depicted that disparity changed through time mostly gradually, following expectations under a Brownian motion model, but also presenting different deviations in the last 4 myr. This suggests that our studied traits evolved in a more complex scenario than that previously pointed out in other works. In fact, complex OU multi-selective regime and BM multi-rate scenarios were the models that fitted best to the morphometric data, in concordance with some previous hypotheses (e.g. ref.^12^).

In this sense, many studies discuss the factors driving the evolutionary diversification of hominoids, and there is an ongoing debate about the tempo and mode of such process. Some authors suggest a gradualistic view of hominid evolution. Freidline *et al*.^39^ studied craniofacial morphological change in Pleistocene hominins as a continuous process that allows them to be separated into distinct temporal clusters, based on the differences and resemblances between their allometric trajectories. On the same direction, Neubauer *et al*.^15^ interpreted that the evolution of the morphology of the modern human brain was marked by a directional and gradual change as a result of the appearance of modern craniofacial morphology and key behavioral innovations. Nevertheless, Du *et al*.^14^ suggested that gradualism in hominid evolution is apparent due to the scale dependence of evolutionary patterns. Using brain size data, they proposed that hominid macroevolution is marked by periods of stasis and/or drift driven by directional selection and other climatic and ecological factors. These factors are believed to have played a key role on hominin evolution^16,40^. Our results conform to a non-gradualistic scenario of hominid cranial evolution, from a macroevolutionary scale. Whilst we see a rather continuous trend in the Miocene, there seem to have been abrupt changes in facial and neurocranial morphology through Plio-Pleistocene times, particularly in the hominoid clade, with an increase in shape diversity between all hominoid subclades. Conversely, the hylobatids probably diversified by random processes, occupying a single adaptive peak through the last 10 myr (Fig. 3).

Within what seems to be a non-continuous process, close to the punctuated equilibrium model, for hominid craniofacial evolution, it is still unclear if these extinct and extant species originated as a result of a single adaptive radiation or successive adaptive radiations due to climate and ecological shifts and/or behavioral and morphological key innovations. Delson and Rosenberger^19^ proposed a pattern of differentiation for Old World monkeys consisting in a set of sequential radiations replacing sister-taxa. In this sense, Foley^12^ suggested the occurrence of distinct successive events with different trends happening at variable rate, rather than a single punctuated radiation event or a continuous process. This author proposed that these adaptive radiations were due to the appearance of adaptive novelties and responses to a shifting environmental context. Furthermore, our morphometric analyses depict that there is a considerable amount of craniofacial size and shape variation in hominoid primates. These results may be an indicator of distinct adaptive radiations as new diverse morphologies might have rapidly evolved with the occupation of emergent niches in a relatively short time^41–43^. Nonetheless, it is noteworthy that if the fossil sample is not representative enough, the gaps in the phylomorphospace that have been interpreted as the occurrence of punctuated events could be just a consequence of missing data.

Specifically, our results partly concur with Foley’s hypothesis as they support the occurrence of successive regime shifts with different rates, but differing in the amount and location of the events that took place. A non-continuous process is largely supported by the fit of OUM and BMM models to our morphometric data (Table 1). Foley^12^ proposed at least five different adaptive radiations, adding two more possible for the Middle and Late Pleistocene hominins (African apes and earliest hominins, early australopithecines, *Paranthropus* species, earliest *Homo* species, larger-brained *Homo* species and finally *Homo sapiens*), while our data-driven SURFACE hypotheses suggest distinct adaptive radiations with regime shifts relative to the origin of each genus (Hylobatidae, *Pan* species, australopithecids plus *Paranthropus* species, and *Homo* species). SURFACE hypothesis estimated using rECV data suggested mainly an adaptive regime involving Hylobatidae family and two other regimes for the first *Homo* species and *H. sapiens*, *H. neanderthalensis* and *H. heiderlbergensis*, respectively (Fig. 3).

What factors could have driven these radiations in the hominid evolution? In the last 10 myr, Eastern and Southern Africa have been through periods of extreme climate variability motivated by global climate shifts (i.e. glaciations, changes in oceanic currents), local effects like volcanic and tectonic changes in the Rift Valley and the dynamics of the lake basins originated by such changes^16,44–48^. During this time, as a result of these climatic shifts, there has been an alternating trend between high tree density and the expansion of grasslands and savanna in the African tropical regions. This is the likely scenario for the moment of divergence between *Pan* and the hominin lineage. Eastern Africa displayed a patchy distribution of forests, woodlands and grasslands, together with altitudinal gradients that configured the appearance of humid and dry habitats in the region^45,47^. This large spectrum niche was occupied ca. 4 myr ago by the generalist lineage *Australopithecus*, which was able to take profit of diverse food resources available at that time along a large geographical distribution, as the fossil record suggests. An expansion of the grasslands may be also associated with a key adaptive trait in this clade: bipedalism, which is also related to changes in neurocranial morphology as the foramen magnum relocated towards a more anteroinferior region in the basicranium (Fig. 1; although this displacement is associated to changes in brain size too)^47,49–51^.

Further, a strong shift to aridification took place around 3 and 2.5 myr, causing an expansion of the savanna in East Africa, corresponding to a peak of a northern hemisphere glaciation and the origins of *Homo* genus. Moreover, numerous cultural innovations took place in Africa during this period, such as the beginning of fire management and the manufacture of lithic artifacts, optimizing the exploitation of resources through, for example, scavenging, hunting and cooking meat^52–57^. Such changes might have allowed an increase in the amount and diversity of consumed resources, together with the occupation of a large range of niches and habitats, within and outside the African continent^47,58,59^.

**Figure 4:**
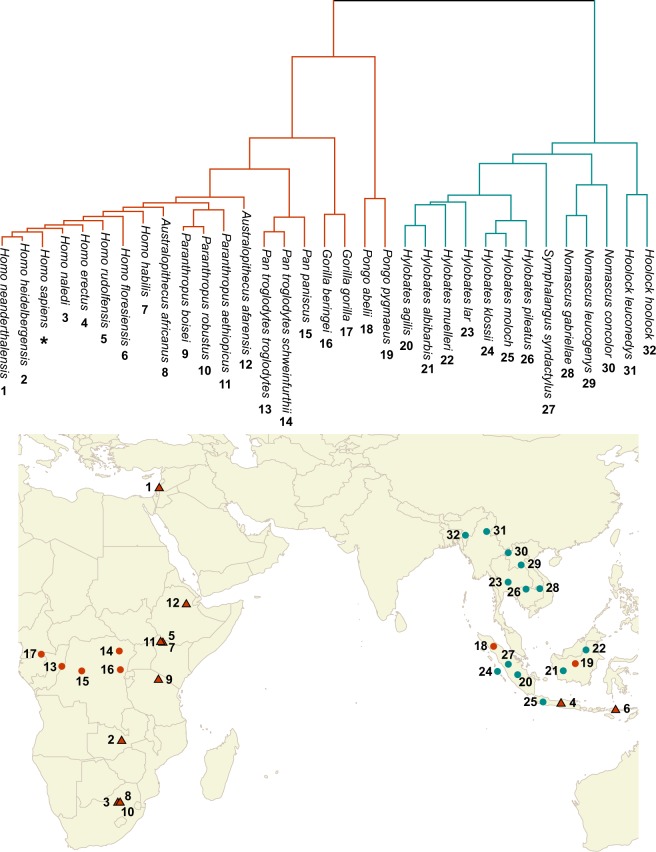
Geographical distribution of the sampled extant (circles) and fossil (triangles) Hominoid species. Distinct colors were used for Hominidae (orange) and Hylobatidae (dark cyan) families. Extant species distribution was obtained from IUCN redlist (www.iucnredlist.org) database, while fossil locations were extracted from bibliographical sources.

Climatic changes and cultural innovations might have been paramount factors related to the hominid morphological diversification, this being caused by or causing them. However, the existence of geographic variation underlying the evolutionary radiation of the hominids (Fig. 4), also possibly related to climate and cultural innovations, suggests that, in some cases within Hominoidea, allopatry might have been one of the main causes for the emergence of new species. In this context, the complexity of factors related to hominid’s radiation suggests that it is an example of a multifarious evolutionary radiation process, including geographic and adaptive factors^43^. However, more paleoclimatic and archaeological data are necessary to establish with confidence the factors driving the observed macroevolutionary pattern.

Summarizing, our results seem to imply that the hominid evolutionary diversification was not a gradual and continuous process, but a more complex and variable scenario involving different evolutionary regimes and factors shifting along the last 10 million years. Although several recent studies have focused on the diversification of the genus *Homo*, our results indicate that, for a better understanding of human evolution, future studies explicitly addressing the complexity of processes at different evolutionary scales are needed. Whereas the small scale patterns are probably linked to microevolutionary processes, the patterns at macroevolutionary scale are probably related to developmental, ecological and evolutionary processes acting across multiple clades^14^. Finally, our results show the importance of adding paleontological data to evolutionary studies exploring macroevolutionary diversifications. Such as previous previous works have demonstrated, the inclusion of fossil variation from periods previous to large extinctions increase our capability for understanding clade evolution^60–63^. In this context it is important to remark that phylogenetic comparative methods using extant species constitute only a preliminary approach to these issues.

## Material and Methods

### Sample

A set of 135 hominoid crania CT scans from twenty-one extant (eight belonging to Hominidae family, thirteen to Hylobatidae family) and 12 fossil species (all of them hominids) was used for carrying out the analyses (Table S1). These scans were obtained from the collections of the Museo de La Plata (MLP), the National Museum of Natural History (Smithsonian Institution; USNM), the Max Planck Institute (MXP), the Royal Museum for Central Africa (RMCA), the Kyoto University Primate Research Institute (KUPRI) and the online repositories African Fossils (africanfossils.org) and MorphoSource (www.morphosource.org).

### Geometric morphometric analyses

In order to analyze and describe extant and fossil species craniofacial shape variation, we employed geometric morphometric techniques. One of the authors (GR) digitized, manually and semi-automatically, 207 three-dimensional landmarks and semilandmarks on.PLY format surfaces obtained from the primate skull CT scans (Figs 1; S3). We explore alternative landmarks and semilandmarks configurations, by example including more surface semilandmarks in the facial skeleton, but the ordination were significantly similar to our final dataset (PROTEST pseudo-correlation = 0.99; P = 0.001; see Fig. S4). These landmarks and semilandmarks were then transformed into shape variables (Procrustes coordinates) through a Generalized Procrustes Analysis (GPA), sliding the semilandmarks with bending energy criterion^28,30^. Thus, variation due to differences in scale, rotation and position of the specimens was eliminated. Shape variables, or Procrustes coordinates, from the consensus of each species were used to perform a Principal Component Analysis (PCA). In addition, centroid size values were used as the size variable. In some cases, fossils showed structural damage and lacked specific cranial structures (mostly zygomatic arches or foramen magnum); this issue was solved by estimating the missing landmarks and semilandmarks with thin-plate spline method using *estimate.missing* function from geomorph^64^ package for R. CT scans were processed by means of 3DSlicer and MeshLab software, landmarks and semilandmarks were recorded using Landmark v3.0^65^ together with geomorph and Morpho^66^ packages for R software. These two R packages were also used in order to perform GPA and PCA analyses.

### Phylogenetic comparative methods

As species originate by a divergence or branching process that can be depicted as a hierarchically structured phylogeny, statistically speaking, they should not be considered as units extracted independently from a same distribution^27^. For such reason, it is necessary to take into account the phylogenetic structure in our analyses. This is the basis of the so-called phylogenetic comparative methods. Here, we use as phylogeny a modified version of the hominoid chronophylogenetic hypothesis used in Grabowsky and Jungers^3^, considering also the fossil-calibrated chronophylogenetic tree obtained by Perelman *et al*.^9^. It is important to remark that the divergence times in our chronophylogenetic tree are based both in Perelman *et al*.^9^ and Grabowsky and Jungers^3^ (modified from Dembo *et al*.^11^), which constrain a Bayesian phylogenetic estimations using the date of first appearance of the fossils.

First, we projected the phylogeny onto the multivariate morphospace obtained by PCA (PC1 and PC2), in order to visualize the morphometric diversity of specific clades (*Homo* genus, Hominidae family, Hylobatidae family). This graph shows clearly how certain clades vary in morphology more than others^32^. This was carried out using *phylomorphospace* function from phytools package^67^ for R. In addition, we mapped and plotted rECV values onto the phylogeny using *contMap* function of phytools package for R software^68^. ECV values for each species were obtained from Isler *et al*.^69^ and Du *et al*.^14^, and the cranial base was defined using a subset of the digitized landmarks and semilandmarks (see Fig. S3). We used the colour-coded values of rECV obtained with *contMap* to visualize the distribution of this trait along the first two components morphospace.

With the purpose of checking the association between the craniofacial shape variation and size, as well as with rECV, we performed PGLS analyses, which consider the expected lack of independence among species by introducing in the error term of the regression a covariance matrix derived from the phylogeny^70,71^. We fitted the PC scores representing more than 90% of total shape variation (PC1 to PC9) to the log-Centroid Size (logCS) through a regression model (PC1-9 ~ logCS), using the package CAPER^72^ for R software.

In order to assess the phylogenetic structure of our data, we ran phylogenetic signal analyses. Firstly, we estimated Blomberg’s^34^ *K* values for PC1-2 and rECV and, secondly, calculated the degree of phylogenetic signal from the set of Procrustes coordinates, obtained from our digitized landmarks and semilandmarks, using the multivariate version of *K* (*K*_*mult*_)^35^. *K* statistic measures the tendency of related species to resemble each other, distinguishing it from random expectations based only on the tree structure and Brownian motion character evolution. *K* values lower that one imply less resemblance between species than expected under a Brownian motion scenario, perhaps due to uncorrelation between adaptive evolution and the phylogeny. While *K* values greater than one should be interpreted as close relatives that resemble to each other more than expected under Brownian motion context^34^. These analyses were performed by means of packages geomorph^64^ and picante^73^ for R.

The patterns of shape (PC1-2), PC90% (PC1-9) and rECV changes within and among the clades along their evolutionary history were depicted by disparity-through-time plots^36^. Disparity is first estimated for the clade as a whole, and then for each subclade defined by a node in the phylogeny. Relative disparity is obtained by dividing each subclade’s disparity values by the total disparity of the clade. Then, mean relative disparity is estimated for every subclade present at the time of each divergence moment. When values are close to zero, it means that the variation is partitioned among the different subclades, while values near to one indicate that a major proportion of the total variation is contained by the subclades^36^. DTT-plots were obtained by using *dtt* function of geiger^74^ package for R.

As previously said, with the intention of assessing which evolutionary processes drove morphological changes in the hominoid clade we tested the fit of a set of evolutionary models to distinct hypotheses. Foley^12^ stated that human evolution was the result of several different events with distinct trends happening at distinct rates (which he interpreted as a series of adaptive radiations), rather than a single punctuated event or a continuous process. The author states that such adaptive radiations (Fig. 3) were mainly driven by the ecology and behavior of hominins, as a response to their environmental context and the appearance of adaptive novelties. We also tested the fit of these evolutionary models to a hypothetical scenario without regime shifts. Particularly, we estimated and compared to our PC and rECV data the fit of single-rate Brownian motion (BM), Early Burst (EB) and single-selective regime Ornstein-Uhlenbeck (OU) processes, together with OU and BM multi-selective regime and multi-rate models (OUM and BMM, respectively; Fig. 3)^31^. These fits were estimated by means of *mvBM*, *mvEB* and *mvOU* functions of *mvMORPH* package^37^. We used Likelihood, simple and corrected Akaike Information Criterion (AIC and AICc, respectively)^75^ to statistically compare the fits of each model to each hypothesis. In addition, we generated data-driven models to better explore the characteristics of the hypothesized discontinuous OU process, employing the SURFACE approach implemented with the *surface* package for R^38^. This technique allows us to estimate a macroevolutionary adaptive landscape for our rECV and PC data, assigning selective regimes to the branches of a tree in forward/backward stepwise phases^26,38^. We finally tested the fit of the previously described evolutionary models to the adaptive landscape hypotheses obtained with SURFACE analyses, and compared these to the two previous hypotheses. All models were fitted to the datasets employing the multivariate functions of *mvMORPH* package^37^.

## Supplementary information

Supplementary Info
