# Supplementary material for: The Evolutionary Radiation of Hominids: a Phylogenetic Comparative Study
Source: Sci Rep. 2019 Oct 24;9:15267. doi: 10.1038/s41598-019-51685-w (PMC6813319; doi:10.1038/s41598-019-51685-w)
Supplement: Supplementary file 1 — Supplementary Info [file 41598_2019_51685_MOESM1_ESM.pdf]

Supplementary information

THE EVOLUTIONARY RADIATION OF HOMINIDS: A PHYLOGENETIC COMPARATIVE STUDY

Guido Rocatti[1] & S. Ivan Perez[1*]

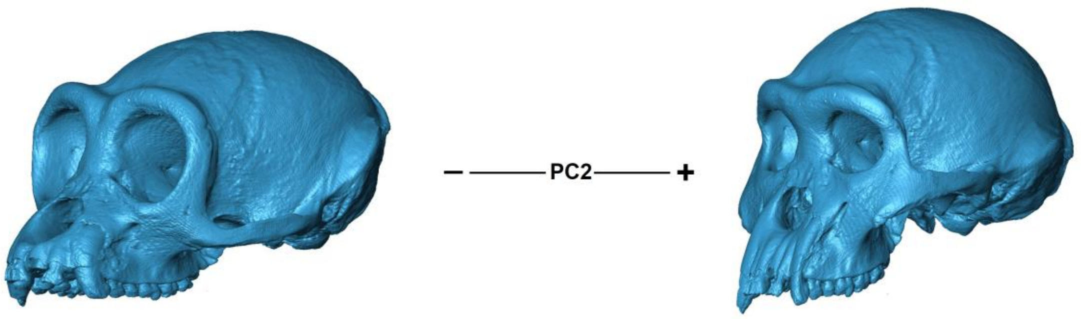

Fig. S1 - Craniofacial shape changes correspondant to PC2 obtained by warping the minimum and maximum scores with Morpho and geomorph packages for R software.

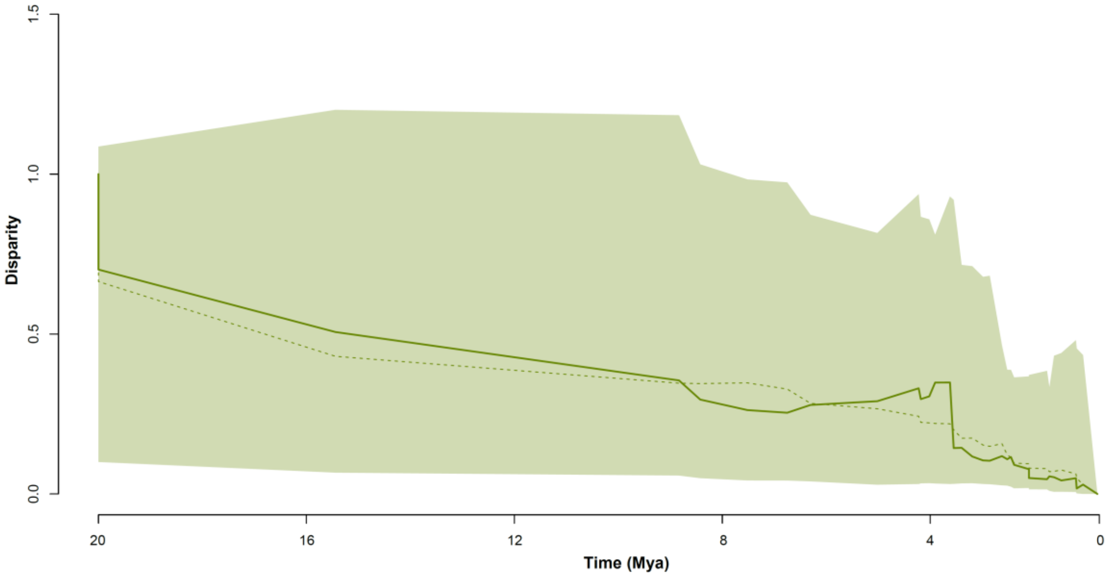

Fig. S2 - Disparity-through-time (DTT) plot using PC1 scores estimated from the hominoid species morphometric data. Dashed line depicts the mean of simulated disparity under a Brownian motion model. The shaded area represents the 95% confidence interval of the simulated data.

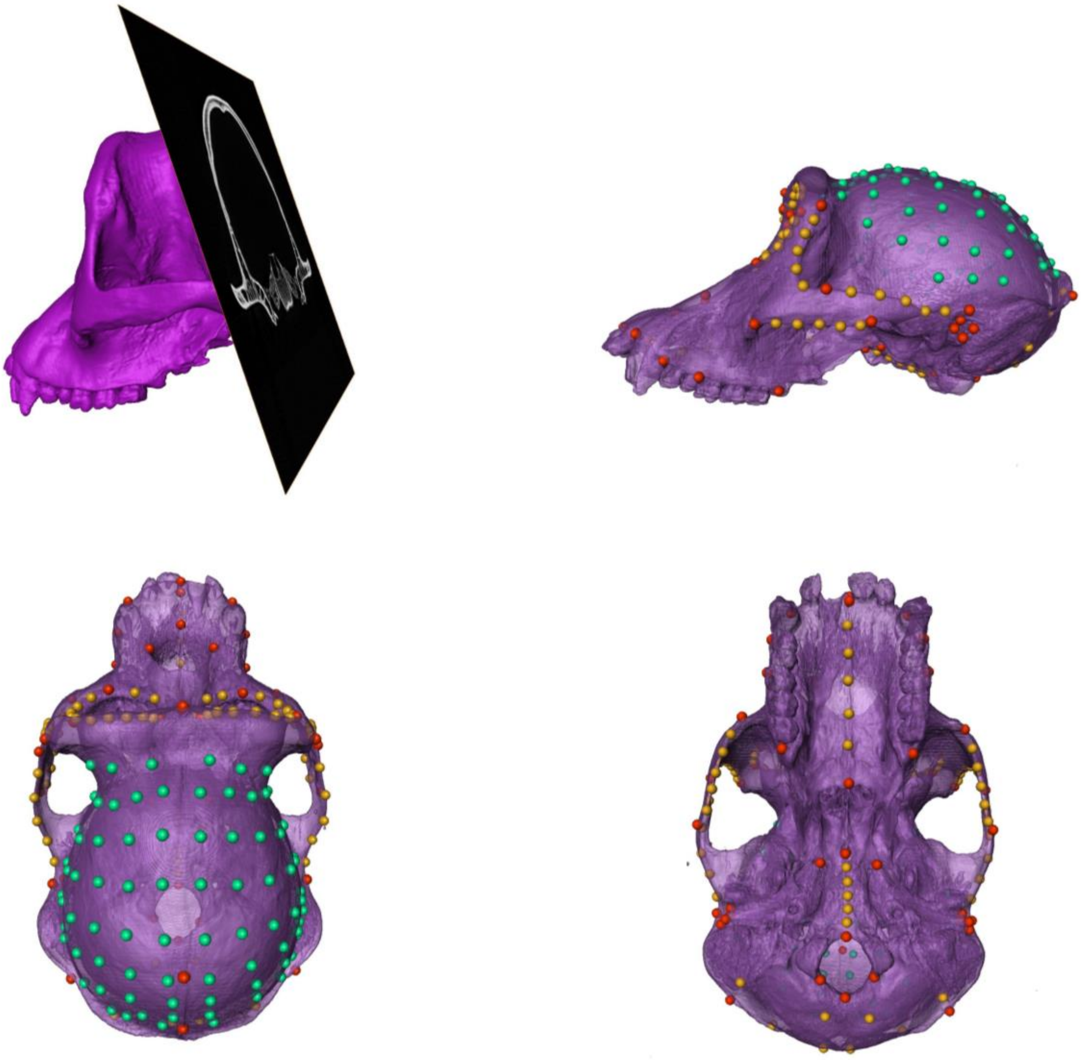

Fig. S3 - (a) Example of CT scan of a chimpanzee (*Pan troglodytes*) skull and one of the slices included in it. (b-d) Lateral, superior and inferior view of .PLY format skull surfaces indicating the location of the studied landmarks (red), curve (yellow) and surface (cyan) semilandmarks.

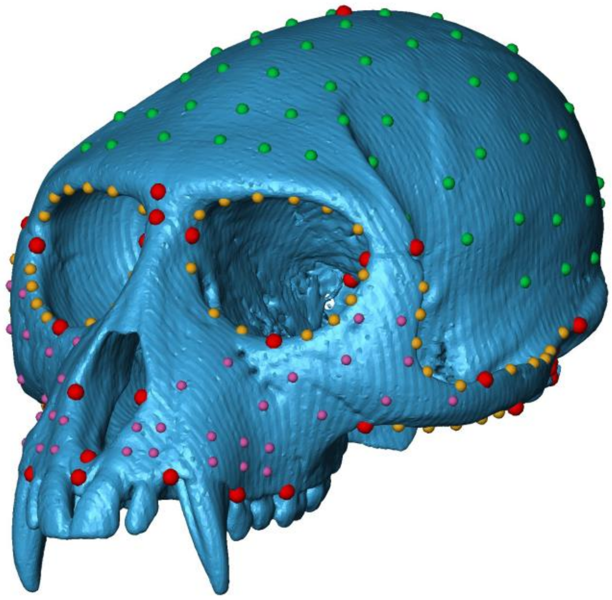

Fig. S4 - CT scan of an African green monkey (*Chlorocebus aethiops*) with landmarks (red), curve semilandmarks (yellow), neurocranial surface semilandmarks (light green) and maxilla surface semilandmarks (pink). Reference point configuration used for comparing alternative landmark surface coverages.

Table S1 - Details of the Hominoid sample used in this study.

**Species**	***n***	**Clade**	**Source or Institution**[a]	**Accesion numbers**
Gorilla beringei	10	Hominidae	USNM	239883; 241232; 395636; 397351; 397353; 545026; 545027; 545028; 545029; 545032
Gorilla gorilla	10	Hominidae	KUPRI; MXP	24 (KUPRI); 109 (MXP); 111 (MXP); 112 (MXP); 128 (MXP); 129 (MXP); 133 (MXP); 146 (MXP); 151 (MXP); 153 (MXP)
Homo sapiens	9	Hominidae	USNM; MLP	698 (USNM); 724 (USNM); 738 (USNM); 840 (USNM); 17735 (MLP); 17737 (MLP); 225129

				(USNM); 226086 (USNM); 228477 (USNM)
Hoolock hoolock	2	Hylobatidae	USNM	257988; 545009
Hoolock leuconedys	1	Hylobatidae	USNM	279146
Hylobates agilis	7	Hylobatidae	USNM	113176; 113177; 113179; 114499; 114501; 123151; 113180;
Hylobates albibarbis	4	Hylobatidae	USNM	145328; 153797; 153798; 153801
Hylobates klossii	4	Hylobatidae	USNM	121674; 121679; 121680; 121681
Hylobates lar	10	Hylobatidae	USNM	83262; 83263; 83264; 83515; 111970; 111988; 111989; 111990; 171982; 253539
Hylobates moloch	2	Hylobatidae	USNM	154721; 154722
Hylobates muelleri	9	Hylobatidae	USNM	83947; 83948; 196779; 196782; 196783; 197643; 198268; 198269; 198270;
Hylobates pileatus	1	Hylobatidae	USNM	201554
Nomascus concolor	2	Hylobatidae	USNM	320786; 320789
Nomascus gabriellae	1	Hylobatidae	USNM	257995
Nomascus leucogenys	2	Hylobatidae	USNM	240490; 240491
Pan paniscus	8	Hominidae	RMCA	13202; 15296; 20881; 20882; 21697; 28712; 29035; 84036
Pan troglodytes schweinfurthii	10	Hominidae	RMCA	83006M13; 83006M14; 83006M15; 83006M16; 83006M17; 83006M31; 83006M32; 83006M33; 83006M34; 83006M35
Pan troglodytes troglodytes	7	Hominidae	USNM	84655; 174699; 174703; 174704; 174706; 174707; 176228
Pongo abelii	10	Hominidae	MXP; KUPRI	210 (MXP); 211 (MXP); 214 (MXP); 227 (MXP); 239 (MXP); 262

				(MXP); 271 (MXP); 272 (MXP); 283 (MXP); 796 (KUPRI)
Pongo pygmaeus	10	Hominidae	MXP	207; 215; 216; 217; 218; 221; 225; 233; 248; 253
Symphalangus syndactylus	7	Hylobatidae	USNM	114497; 141160; 141161; 143577; 143580; 171981; 271048
Homo habilis†	1	Hominidae	AfricanFossils.org	KNMER-1813
Homo erectus†	1	Hominidae	MorphoSource/Duke University	Sangiran 17 (EA-CCC-06)
Homo neanderthalensis†	1	Hominidae	MorphoSource/Duke University	Amud 1 (EA-CCC-08)
Homo heidelbergensis†	1	Hominidae	MorphoSource/Duke University	Kabwe skull (EA-DCC-03)
Homo naledi†	1	Hominidae	MorphoSource/Duke University	Composite skull based on DH1 and DH3.
Homo rudolfensis†	1	Hominidae	AfricanFossils.org	KNMER-1470
Homo floresiensis†	1	Hominidae	Human Evolution Gallery – Natural History Museum (London)	LB-1
Australopithecus afarensis†	1	Hominidae	MorphoSource/Duke University	Composite cranium based on AL 333
Australopithecus africanus†	1	Hominidae	European Virtual Anthropology Network (EVAN)	STS-5
Paranthropus boisei†	1	Hominidae	MorphoSource/Duke University	OH5 (EA-CCC-02)
Paranthropus aethiopicus†	1	Hominidae	AfricanFossils.org	KNM-WT 17000
Paranthropus robustus†	1	Hominidae	European Virtual Anthropology Network (EVAN)	SK-48
**TOTAL**	**138**			

[a] Abbreviations defined in text.
